# What the Public Was Saying about the H1N1 Vaccine: Perceptions and Issues Discussed in On-Line Comments during the 2009 H1N1 Pandemic

**DOI:** 10.1371/journal.pone.0018479

**Published:** 2011-04-18

**Authors:** Natalie Henrich, Bev Holmes

**Affiliations:** 1 Centre for Health Evaluation and Outcome Sciences, Providence Health Care, Vancouver, Canada; 2 School of Population and Public Health, University of British Columbia, Vancouver, Canada; 3 Michael Smith Foundation for Health Research, Vancouver, Canada; Dana-Farber Cancer Institute, United States of America

## Abstract

During the 2009 H1N1 pandemic, a vaccine was made available to all Canadians. Despite efforts to promote vaccination, the public's intent to vaccinate remained low. In order to better understand the public's resistance to getting vaccinated, this study addressed factors that influenced the public's decision making about uptake. To do this, we used a relatively novel source of qualitative data – comments posted on-line in response to news articles on a particular topic. This study analysed 1,796 comments posted in response to 12 articles dealing with H1N1 vaccine on websites of three major Canadian news sources. Articles were selected based on topic and number of comments. A second objective was to assess the extent to which on-line comments can be used as a reliable data source to capture public attitudes during a health crisis. The following seven themes were mentioned in at least 5% of the comments (% indicates the percentage of comments that included the theme): fear of H1N1 (18.8%); responsibility of media (17.8%); government competency (17.7%); government trustworthiness (10.7%); fear of H1N1 vaccine (8.1%); pharmaceutical companies (7.6%); and personal protective measures (5.8%). It is assumed that the more frequently a theme was mentioned, the more that theme influenced decision making about vaccination. These key themes for the public were often not aligned with the issues and information officials perceived, and conveyed, as relevant in the decision making process. The main themes from the comments were consistent with results from surveys and focus groups addressing similar issues, which suggest that on-line comments do provide a reliable source of qualitative data on attitudes and perceptions of issues that emerge in a health crisis. The insights derived from the comments can contribute to improved communication and policy decisions about vaccination in health crises that incorporate the public's views.

## Introduction

In March 2009 the first cases of H1N1 influenza were reported in Mexico. In June 2009, the World Health Organization (WHO) declared a pandemic [1] and discussions commenced worldwide among public health officials, governments and pharmaceutical companies about development of an H1N1 vaccine. By later that summer, production of the new vaccine had begun with the goal of producing both the seasonal and new H1N1 flu vaccines in time for the upcoming annual influenza season. While health and political officials hoped that demand for the vaccine would be high, polls indicated that as much as 50% of Canadians did not intend to get vaccinated [2]; actual vaccination rates during the pandemic were estimated at approximately 41% of Canadians [3]. As health officials struggled to convince the public of the safety and value of vaccination against H1N1, the question loomed large: how could such a mismatch exist between the officials' expectations about the public's acceptance of the vaccine and the intentions to vaccinate voiced by the public? It appeared that the factors that officials perceived as relevant in the decision making process differed from those perceived by large segments of the Canadian public. As well, the information provided to the public about the vaccine did not resonate with them and in many cases was being rejected.

In light of this discordance between public and official attitudes towards new vaccines developed for use in a crisis situation, it became important to better understand how the public perceives novel vaccines developed and promoted during an outbreak, and what factors influence their decision making. Prior to the H1N1 pandemic, little was known about attitudes towards *new* vaccines for *new* diseases that are developed for use by children and adults (but see [4]–[5]); as is the situation during a pandemic. With the development of the H1N1 vaccine, and polls indicating low public acceptance, several surveys were rapidly conducted to identify public attitudes about the new vaccine and their intended uptake of the vaccine [6]–[13]. These surveys provided important information on levels of concern about H1N1 and the H1N1 vaccine, the association between seasonal flu vaccine uptake and intended uptake of H1N1 vaccine, and intentions to vaccinate.

The surveys, however, were not able to provide qualitative insight into public attitudes or allow for open-ended, respondent-driven discourse about the H1N1 vaccine. Qualitative health research is necessary because it identifies how people understand and experience situations or issues and provides understanding into why people have these perspectives [14], and therefore can be used to address health issues more effectively [15]. To date, qualitative studies have required a time consuming form of data collection that has not been conducive to a crisis situation. Now, with the increasing popularity of the internet [16], there is a new opportunity that allows for insight into the attitudes, concerns, and deliberation processes of the public in real time during a health crisis, which can contribute to a participatory approach to research and increase the relevance of public health to the public [17]. Most media outlets in Canada provide news online, and many offer readers the option to comment on specific stories and to respond to other readers' comments. These comments and responses, often functioning like a conversation among readers (who include lay people as well as topic experts), serve as a gauge of public opinion that is immediate, spontaneous and (presumably) honest. They contain a wealth of information about how people think and feel about the topic at hand as well as how they react to, and the extent to which they agree with, others' views. Manosevitch and Walker ([18]:5) suggest that such comments “provide more diverse and authentic public deliberation” than traditional letters to the editor because they reflect readers' immediate responses, provide the readers unlimited space, are unedited, and are censored only in that offensive comments are removed.

With much social science research, study participants provide information in response to specific questions (and possibly on a topic about which the participants have no real interest or opinion). In contrast, data derived from comments are entirely participant driven and can include content unexpected by the researchers that is valuable in that it reveals the issues that matter to the commenters; people engage unsolicited in commenting so we can presume that the topics on which they write are important to them. As well, comments reflect not only the writers' ideas about a particular news article but of the wider “story” circulating in public discourse and, as such, they provide insight into public opinion about an issue in its entirety. Learning about public attitudes from publicly available on-line comments can also be a cost-effective and rapid way to collect data. To the best of our knowledge, comments posted in response to news articles have been used to date as a data source in only a handful of studies [19]–[22].

The representativeness of posted comments cannot be known because information on the demographics or socioeconomics of the commenters is unavailable [19]. However, as Rowe et al. ([19]:366) indicate, the comments are still a valuable source of research data because 1) the content is immediately available and reflects the “respondents' current attitudes rather than remembered opinions generated after the fact,” 2) comments are potentially available from very large numbers of respondents, and 3) commenters may be similar to participants in other types of studies in that, like other participants, they include those people who are inclined to share their opinions. Additionally, even if the comments are not representative, they do reflect the perspectives of a large segment of the population and are thus worthy of study.

Our study had two objectives. First, we were interested in understanding the Canadian public's attitude towards the H1N1 vaccine during the pandemic, and the aspects of the vaccine that were impacting their decision making regarding uptake of this vaccine. Second, we wanted to assess the extent to which on-line comments can be used as an informative and reliable data source to capture real-time public attitudes during an emerging health crisis. To this end, we analysed comments posted in response to news articles related to the H1N1 vaccine from three major Canadian news sources. Validity in qualitative research consists of producing a sound, coherent, convincing argument that is grounded in and supported by the data. Claims can be verified by references to other studies [23]and by showing textual material used in the analysis, allowing others to assess the researcher's interpretations [24]. Consistent with this concept of validity, we provide exemplary comments associated with the key themes in the comments and comparisons to related studies. Our findings identified and provided context for understanding public perceptions and concerns about the H1N1 vaccine and suggest that on-line comments are a valuable source of qualitative data.

## Methods

### Reader demographics

Comments were included from three Canadian on-line news sites: globeandmail.com [GM], vancouversun.com [VS] and cbc.ca [CBC]. The demographics of commenters are not known but reader profiles are available from each site, which provides information on the population from which commenters are drawn. Table 1 provides a breakdown of readers by age, sex, household income and number of unique visitors per month.

**Table 1:** Reader profile for vancouversun.com, globeandmail.com and cbc.ca.

Category	Subcategory	Vancouversun.com^1^	Globeandmail.com^2^ *	CBC.ca^3^
Sex	Female	47%	36%	50%
	Male	53%	64%	50%
Age	Under 25	16%	5%	15%
	25–34	23%	17%	18%
	35–44	20%	19%	21%
	45–54	21%	25%	22%
	55+	21%	34%	24%
Region	Atlantic	4%	6%	12%
	Quebec	5%	3%	7%
	Ontario	31%	67%	44%
	Prairies	13%	12%	20%
	BC	47%	12%	17%
Household Income	$75k+	37%	n/a	45%
	$100k+	18%	53%	n/a
Number of unique monthly visitors**		1,300,000	6,000,000	5,800,000

*globeandmail.com regional readership information is based on readership of the Globe and Mail print weekday newspaper. This information is not available for their on-line news readers.

**All data on number of unique monthly visitors was taken from Globe and Mail: About our digital network.

1 Reader profile information taken from Vancouver Sun Advertising Plan Book (2010).

2 Reader profile information taken from Globe and Mail: About our digital network, Globe and Mail: 2011 Media Kit, Globe Website User Profiling Study (2010), and Globe Readership and Circulation (2009).

3 Reader profile information taken from CBC.ca audience profile (2010).

### Article selection

A total of 12 articles were selected from the GM, CBC and VS websites (Table 2). Article selection was based on an on-line search that identified all articles posted between March 2009 (the time of the first identified case of H1N1) and May 2010 (the end of the Canadian influenza season) containing the words *H1N1* and *vaccine*. The three articles from each news source that received the most comments were selected for inclusion in the study. We assume that readers respond to articles that resonate with them and hence the number of comments posted in connection with an article is a measure of the importance of the article to readers.

**Table 2:** Articles included in the study.

News source	Date	Headline	Total # of comments*	# of comments included in analysis**
CBC	November 6, 2009	H1N1 overplayed by media, public health: MDs	847	202
CBC	June 11, 2009	WHO declares swine flu pandemic, no change in Canada's approach	348	206
CBC	August 6, 2009	Canada to order 50.4 million H1N1 vaccine doses	674	201
CBC	April 29, 2009	WHO boosts pandemic alert level to 5	754	201
CBC	April 30, 2009	Canada doing all that's needed to respond to swine flu: PM	285	202
Vancouver Sun	October 24, 2009	Column: Swine flu shot? Not for this little piggy	161	151
Vancouver Sun	October 26, 2009	Urban myths about the H1N1 vaccine	70	64
Vancouver Sun	November 18, 2009	Canucks jumped the H1N1 vaccine queue, health officer says	108	103
Globe and Mail	August 12, 2009	A summer of discontent over Ottawa's flu plan	117	62
Globe and Mail	October 23, 2009	Health officials scramble to counter H1N1 myths	296	137
Globe and Mail	November 1, 2009	Health officials caught off guard by demand for H1N1 shot	268	140
Globe and Mail	November 2, 2009	MPs debate H1N1 vaccine rollout	265	126

*The total number of comments includes **all** comments posted for an article.

**The number of comments included per article in the analysis differs from total number of comments for that article because only one comment per commenter was included and approximately 200 comments were randomly selected for inclusion for articles with more than 200 comments (after removing multiple comments from the same commenter).

An additional three articles were selected from time periods that were underrepresented in our sample (spring and summer 2009); articles were selected based on the highest number of comments per article during the specified time period.

Our database included the comments for each article and the commenter's username. If a commenter posted more than one comment for an article, we included only the first comment. We assume that by allowing only one comment per user name per article that we are including one comment per commenter. This is based on the assumption that readers use a single username when posting on a given news site[19]; this is likely given there is no limitation on the number of comments an individual can post so a person can use a single username to post unlimited comments. When the number of comments per article exceeded 200, 200 comments were randomly selected for inclusion in the database. All comments were imported into *NVivo 8* (QSR International), a qualitative analysis software, for coding and analysis.

### Data set

Comments were available on each news site via a link next to the article. Comments were displayed with the commenter's username and the date and time that the comment was posted. For each of the targeted articles every comment and username was copied and pasted into an Excel database. Comments could be of any length, and were not screened for spelling, grammar or accuracy. An example of a comment as it would appear on-line is:

***alta45*** **wrote:**

The press is totally out of control on this one. They seem to need to do that all the time nowdays. Its the flu wash your hands take the regular precations. The sky is not falling.

Agree Disagree Policy Report abuse

Posted 2009/05/01 at 9:08 AM ET

To access the comments, the article headlines provided in Table 2 can be entered into the search engine at the corresponding news site; however articles and their comments are archived and inaccessible on-line after a period of time determined by each of the news sites. Comments that are no longer on-line are available from the corresponding author.

### Coding

A set of themes was developed to capture the topics and issues that people were commenting on related to the H1N1 vaccine. The themes were developed using a combination of approaches. Deductively, we based 12 themes on previously conducted focus groups that examined attitudes and concerns about new vaccines for use in a pandemic [5]. An additional two themes emerged from the comments based on the evident recurrence of these themes in the comments. Many ideas and opinions were expressed in the comments that were not reflected in the themes because they were unique or rare; the themes that emerged from the comments only include ideas/opinions that appeared repeatedly across comments. Three themes were created based on issues we felt may be relevant given the situation and coverage surrounding the pandemic; these codes related to comments about the WHO (which was responsible for declaring H1N1 a pandemic) and confusion related to information conveyed about the disease and the vaccine, which we thought may have been an issue given changing or ambiguous information released to the public about things such as who was high priority for vaccination and whether pregnant women should use adjuvanted vaccine. Table 3 indicates which themes were developed with each approach. Codes were created for each theme, with codes reflecting directionality of a comment (i.e., whether it was a positive or negative statement).

**Table 3:** Percentage of comments containing each theme and code.

Themes^a^ (% of ALL comments)	Codes (% of comments WITHIN a theme)
Fear of H1N1 (18.8%)	Low fear (63%)
	High fear (37%)
Responsibility of media (17.8%)	Media responsible (15%)
	Media irresponsible (85%)
Gov't competency (17.7%)	Gov't competent (21%)
	Gov't incompetent (79%)
Gov't trustworthy (10.7%)	Gov't trustworthy (3%)
	Gov't untrustworthy (97%)
Fear of H1N1 vaccine (8.1%)	Low fear (27%)
	High fear (73%)
Pharmaceutical companies (7.6%)	Pharmaceutical companies (100.0%)
Personal protective measures (5.8%)	Personal protective measures (100.0%)
Gov't general statements^b^ (4.4%)	General statements- positive (9%)
	General statements- negative (91%)
Media trustworthy (4.2%)	Media trustworthy (5%)
	Media untrustworthy (95%)
Anti-vaccines (3.8%)	Anti-vaccines (100.0%)
World Health Organization (WHO)^e^ (3.2%)	WHO – positive (28%)
	WHO – negative (72%)
Author^b^ (3.1%)	Author – positive (38%)
	Author – negative (63%)
Public good (3.0%)	Public good (100.0%)
Equity (2.1%)	Equity (100.0%)
Individual choice (2.1%)	Individual choice (100.0%)
Confusing information^c^ (1.1%)	Confusing information (100.0%)
Feeling confused^c^ (0.6%)	Feeling confused (100.0%)

a Unless otherwise indicated, themes were derived from previously conducted focus groups that examined attitudes and concerns about new vaccines for use in a pandemic.

b These themes emerged from the comments.

c These themes were created based on issues the authors felt may be relevant given the situation and coverage surrounding the pandemic.

As is standard procedure for qualitative coding of text, comments were coded based on the coder's assessment of the themes conveyed in each comment. In order to minimize the effect of coder subjectivity, 10% of the comments from each article were coded by two coders. Agreement between coders was extremely high and any discrepancies were resolved by consensus. Discussion of the reason for the discrepancies also allowed for improved consistency in how the themes should be applied.

### Analysis

The frequency of themes was calculated in aggregate across all articles, for each source, and for each time period: spring (April), summer (June and August), fall (October and November). The discontinuity in dates occurs because, based on our inclusion criteria, no articles were selected from May, July, September or after November. The comments within each theme were reviewed and when the same ideas were frequently repeated across comments then these ideas were classified as **subthemes**. Exemplary quotes that best represented a theme or subtheme were selected.

This study did not require ethics approval because all data were publicly available and anonymous.

## Results

We selected 12 articles to include in the analysis; for each article, the news source, headline, date of publication, total number of comments posted for the article, and the number of comments included in the analysis from that article are provided in Table 2. A total of 1,796 comments were included in the analysis; 1,013 from CBC, 465 from GM and 318 from VS. Due to an error in the random selection of CBC comments, between 201 and 206 comments per article were randomly selected for inclusion rather than 200, thus leading to the inclusion of an extra 13 comments. The coding and analysis process resulted in a final set of 17 themes and 26 codes (Table 3). The percentage of comments that contained each of the themes and codes are presented in Table 3. Subthemes were identified for the themes mentioned in at least 5% of the comments (Table 4).

**Table 4:** Subthemes for themes mentioned in at least 5% of comments.

Theme	Subtheme
Fear of H1N1 (low)	Few deaths have been caused by H1N1
	H1N1 is not different from seasonal flu
	Seasonal flu is more deadly than H1N1
Fear of H1N1 (high)	H1N1 is a new disease
	Risk of mutation
	Young adults are dying
	High mortality and/or severe morbidity
Government competency (incompetent)	General incompetence
	Government took wrong or inadequate measures
	Prime Minister and public health authorities blamed
Government trustworthiness (untrustworthy)	Government motivated by politics
	Government motivated by economic/financial incentives
	General mistrust of government
Fear of H1N1 vaccine (high)	Fear of adjuvants
	Fear of mercury
	Insufficient testing and lack of information about side effects
	Mistrust of government claims about vaccine safety
Fear of H1N1 vaccine (low)	Risks of H1N1 greater than risks of the vaccine
	Vaccine is safe
Pharmaceutical companies	Government financially supporting pharmacy industry
	Government protecting pharmaceutical industry
	Pharmaceutical companies benefit from the pandemic
Personal protective measures	Basic prevention
	Diet/dietary supplements

How often themes were mentioned in the comments provides a guide to the themes that are likely to be most important to commenters. Consequently, the following is an exploration of themes mentioned in at least 5% of the comments (i.e., at least 90 comments) as these may be the themes that commenters cared about most and hence which may have been more likely to influence vaccination decisions. Exemplary quotes are included for each theme and they are presented unedited and in italics. Following the discussion of the key themes, we describe how frequency of themes varied over time and by news source.

### Fear of H1N1

Low and high fear of H1N1 was expressed, although there were nearly twice as many comments reflecting low fear (212) compared to high fear (125). Three subthemes characterized the reasons for a low fear of H1N1.

#### 1. Few deaths have been caused by H1N1

Given the relatively low rate of mortality caused by H1N1, commenters expressed little concern about the disease. They compared H1N1 to other diseases and causes of death with substantially higher mortality rates and concluded that H1N1 was sufficiently lacking in severity that it did not warrant heightened concern.

*“So we had the first wave of flu in the spring and the entire southern hemisphere went through their flu season and out of 6 billion people, 5,000 have died? That is like the number of children who die from bad water in 10 hours. 25 million have died from AIDs yet no one ever considered mandatory testing or quarantines when that pandemic first started. What makes H1N1 so special?”* [GM October 23]

#### 2. H1N1 is not different from seasonal flu

Commenters characterized H1N1 influenza in a generic “flu” category and did not differentiate it from seasonal flu. Because many people do not perceive seasonal flu as serious, H1N1 was also not perceived as serious.

*“A flu travels the world every year and kills thousands in Canada… So far it appears the only difference here is this one spent some time in some pigs.”* [CBC April 29]

#### 3. Seasonal flu is more deadly than H1N1

Commenters compared H1N1 to seasonal flu and perceived seasonal flu as more deadly than H1N1. They concluded that since people do not have elevated fear about seasonal flu then there should not be elevated fear about a less serious form of influenza.

*“I won't be getting the shot. I'm pro-vaccine though. For things that are actually dangerous. Not something that is far less lethal than normal flu.”* [CBC August 6]

In contrast to comments reflecting low fear of H1N1, other comments indicated high fear. Four subthemes emerged that characterized reasons for fearing H1N1.

#### 1. H1N1 is a new disease

Commenters were concerned about the newness of H1N1 because they feared that humans lack immunity to a new viral strain.

*“The H1N1, by numbers alone, is most certainly not as devastating as the seasonal flu….But the reality is that more people will get it since far fewer people have immunity.”* [VS October 26]

#### 2. Risk of mutation

Although H1N1 in its current form was not necessarily feared, commenters feared that H1N1 would mutate into a much deadlier virus. Their fear was for this potentially new form of H1N1.

*“Sure, the H1N1 virus may not seem like much now, but what they're especially afraid of is this; When a cell is infected with one flu virus, and then a SECOND virus infects the same cell, you get a combination virus, and when that happens there's no real way of telling what you're going to get. If for example the H1N1 virus were to infect someone with SARS and the viruses combined, it's entirely possible that you'd get a new 1918 flu out of the deal, and then you'd have to start worrying about where to bury the people living in your apartment block. That's what the fuss is about.”* [CBC November 6]

#### 3. Young adults are dying

H1N1 was seen as distinct from seasonal flu in that healthy, young adults were dying; hearing that a segment of the population perceived as less vulnerable to illness was succumbing to the disease increased fear about H1N1.

*“I think it's important to remember that even healthy people with no underlying health conditions have become SERIOUSLY ill and have died from the H1N1 virus.”* [VS October 24]

#### 4. High mortality and/or severe morbidity

Some commenters were highly concerned about the health impact of H1N1, believing that it widely causes severe illness as well as posing a significant risk of death.

*“you refer to H1N1 as mild like the flu, well may be you should tell that to the family of the 13 year old boy from toronto who lost his life after having symptoms for 2 days or to the 3 year old girl in my town who now lay in hospital in critical condition and her family who is watching her die, or maybe the parents of 89 children from the high school in my small town, who are having to coranten [quarantine] their homes and fight for there children to get better,or how about the 48 student in my childrens public school who are severly sick,most of these children healthy before h1n1.…I think once healthy children start to die, its time to take action.”* [VS October 24]

### Responsibility of media

This theme captured comments about how well the media performed its job in reporting on the H1N1 pandemic. It included statements regarding whether news coverage was factual and accurate, whether the stories were covered in sufficient depth, and whether the right stories were being covered. Commenters generally characterized the media as irresponsible (260 comments) with only 45 comments crediting the media with responsible reporting. The primary criticisms of the media were that reporting lacked context and facts.

*“CBC - could you possibly provide us with a little perspective? There are many flus that circulate the globe every year, and kill many more people than swine flu has. I understand that perhaps what differentiates this one is that it is "new". However, could you maybe provide us with stats as to how many people have died from "the flu" in Canada during this period in which 4 people have died from swine flu? thanks.”* [CBC June 11]

*“There is so much misinformation out there right now- and this article with no sources or references is only contributing to it. Shame on you Vancouver Sun!!!”* [VS October 24]

The positive comments about the media's reporting of the pandemic credited the media for the abundant coverage of the situation and providing the facts needed to enable people to make their own decisions about H1N1.

*“… how is that so many of us know that regular seasonal flu is more severe than H1N1? How? We read it in the media!”* [CBC November 6]

### Government competency

The government competency theme captured comments that addressed the government's ability to deal with the H1N1 pandemic and included comments targeting any level of government. Two hundred and fifty-two comments were critical of the government and 66 comments were positive. Three subthemes emerged regarding the government's incompetency.

#### 1. General incompetence

The government was portrayed as being generally incompetent, with the mishandling of the pandemic being just one more example illustrating its ineptitude. Comments included both criticisms specifically about the pandemic, as well as comments about overall maladroitness.

*“This is outrageous and quite frankly, unbelievably negligent. They spent months scaring the heck out of the public, urging all of us to get vaccinated. So now, their excuse for being woefully disorganized is they didn't expect so many people to show up? They made their plans on the assumption people wouldn't listen to them? This is the logic officials trusted with protecting our lives are using? Shocking. Disgraceful. When are these incompetent health officials going to be held accountable? How many people are going to die because of organizational incompetence?”* [GM November 1]

#### 2. Government took wrong or inadequate measures

The government was accused of taking too little action to prevent and control the pandemic, as well as taking wrong actions, especially with regard to the handling of the H1N1 vaccine in terms of acquisition, promotion and dispensing of the vaccine.

*“I fail to see why the government bought 50.4 million vaccines for a country with a population of about 35 million of which likely less than 50% will get vaccinated.”* [CBC August 6]

#### 3. Prime Minister and public health authorities blamed

For many commenters, the ultimate responsibility for the perceived poor handling of the pandemic lay at the feet of Canadian Prime Minister Stephen Harper. He was criticized both for his actions (for example, that he should have ordered vaccine from more than one supplier) as well as his inaction (for example, that he should have imposed travel restrictions). Others held the public health authorities responsible for the mishandling of the situation.

*“Is this the best our public health authorities can do (at all levels of government) especially given the long planning timelines (we knew a second wave H1N1 influenza was coming) and this particular influenza outbreak has not been ‘virulent’ in terms of its impact? How would our public health authorities respond in an emergency e.g. terrorist incidents involving IEDs, "dirty" bombs, biohazards etc.?”* [GM November 2]

A minority of commenters (66) indicated the government handled the situation competently. In direct opposition to the charges of ineptitude, these commenters praised the response as appropriate, felt that Prime Minister Harper and the public health authorities did their jobs well, and were satisfied with the overall handling of the vaccines. Examples of these comments include:

*“There's nothing to defend. They have done a very good job of getting the vaccine out, educating people and making sure that when all is said and done every Canadian will receive a free vaccination if they want one. You can't do much better than that. Good work Canadian government.”* [GM November 2]

### Government trustworthiness

This theme captured comments about whether or not the government was trusted to be dealing with the pandemic in the best interest of the public. Distinct from the ability of the government to handle the situation, comments included in this theme dealt with the *motivations* and *intentions* of the government. Of the 193 comments on government trustworthiness, 188 comments conveyed mistrust. There were three primary reasons why commenters indicated they did not trust the government's handling of the pandemic.

#### 1. Government motivated by politics

Rather than making decisions about the pandemic that would most benefit the public, commenters believed that politicians' decisions were driven by political motives. These motivations included a desire to improve their own popularity and, conversely, to hurt the opposition. Both the majority and opposition parties were perceived to be acting politically.

*“…its embarassing that a government would put their own political ambitions ahead of the health of their citizens, it really pisses me off.”* [GM November 1]

#### 2. Government motivated by economic/financial incentives

Commenters expressed the opinion that the government's handling of the pandemic was largely influenced by economic considerations. In some cases, commenters felt that the government was making decisions to try and protect the economy, such as promoting vaccination in order to minimize workplace absenteeism. However, these decisions were not perceived as necessarily being aligned with the public's health interests. Other commenters believed that decisions were being made as a consequence of pressure from pharmaceutical companies and that collusion between government and these companies was driving policy.

*“Keep in mind that the top political priority (indirectly) will always be "mitigating the impact on the economy".”* [CBC April 29]

#### 3. General mistrust of government

Regardless of what actions were taken by the government, commenters indicated that some people would always be suspicious of the government's motives because of a general mistrust of government. The mistrust did not stem from the handling of the pandemic; rather it carried over to this situation and especially impacted perceptions of the H1N1 vaccine.

*“Governments are run by politicians. When politicians speak to the people they are usually lying and trying to swindle you into something. Now when they are asking you to take an injection, who can blame folks for being suspicious?”* [GM October 23]

### Fear of H1N1 vaccine (low/high)

This theme captured comments related to concerns about the H1N1 vaccine; whether commenters' level of concern was high or low, and the reasons for their concern or lack thereof. There were substantially more comments indicating high concern than low concern (106 vs.39 comments). Four main concerns contributed to high fear of the vaccine.

#### 1. Fear of adjuvants

Commenters were highly suspicious of the H1N1 vaccine adjuvant, squalene. Concerns centred around the limited testing of squalene and the uncertainty about the side effects that could emerge when large numbers of people are exposed (effects that may not have been detected in small clinical trials) and effects that could develop in the long term.

*“The swine flu vaccine however is problematic because it uses an adjuvant, squalene which has been linked in numerous studies to causing autoimmune diseases in animals. It has been used in Europe for a couple of years now but only on elderly patients. So there is little testing and track record on the additive. Why on earth did we order one with an unapproved additive that has gone through limited testing when it was possible to make one with out using a form of vaccine that is more common and has more years of testing behind it?”* [VS October 26]

#### 2. Fear of mercury

Commenters feared taking a vaccine that included mercury, which is known to be dangerous to humans. They argued that if it is unsafe to be exposed to mercury from other sources then it can't be safe to be exposed to mercury in the vaccine.

*“I'll pass on the mercury/toxin cocktail. Thanks anyhow.”* [CBC August 6]

#### 3. Insufficient testing and lack of information about side effects

A main cause of fear about the vaccine stemmed from what was perceived to be insufficient safety testing. Commenters expressed concern about small samples in the clinical trials, as well as trials that were too short to determine long-term effects. Commenter were not arguing that the vaccine was unsafe, but rather that we lacked information needed to conclude that it was safe. Commenters expressed that the Canadian public would be guinea pigs and that the real safety trial was being conducted on the masses.

*“I wonder if GlaxoSmithKline is already producing prescription drugs to deal with the side-effects of the vaccine. Get this, "Results from trials of the avian flu vaccine suggest one dose should be enough"! Would you want to take something that research has "suggested" "should" work? Or that gets only two months of clinical trial (remember, long-term is where cancer etc etc shows up!) Or that will be monitored "closely when it comes on to the market, and if there are any concerns, they'll be addressed". Essentially, the vaccine hasn't been properly tested, they know it, they'll be addressing problems as they go and people get sick… Canadians will be the gunieau [guinea] pigs for this vaccine. Ridiculous!”* [CBC August 6]

#### 4. Mistrust of government claims about vaccine safety

A final concern, though less frequently mentioned than the other subthemes, related to mistrust of government (see above) and the government's claims about vaccine safety. Commenters expressed that the claims could not be trusted because the government was not trustworthy.

*“The Guberment says its safe, so of course it MUST be safe. Go on, get your injection, after all the guberment says it safe. How does it feel to be a human experiment? anyone that believes whatever the government tells them deserves the complications of the vaccine. stupid sheeple.”* [VS October 26]

Overall, commenters who had either low, or no, fear of the vaccine tended to express one of two beliefs: that the risks of H1N1 were greater than the risks of the vaccine or that the vaccine was safe.

*“To summarize, nothing is risk free but if you do your homework in an objective and unbiased way you will find that the risk of H1N1 is well documented and thousands fold (at least) higher than any likely risk from vaccination.”* [VS October 26]

*“They make a new flu vaccine every year, and it doesn't need years and years of testing because it's the same basic and proven formula, they just change the strains that it protects you from, as they try to predict each year's flus. This is the same technology.”* [CBC August 6]

### Pharmaceutical companies

This theme included comments about pharmaceutical companies that developed the H1N1 vaccine. All of these comments were highly critical, and largely fell into the following three subthemes.

#### 1. Government financially supporting pharmacy industry

Criticism was expressed that the reason the government undertook the H1N1 vaccination campaign was because it generated windfall earnings for the pharmaceutical companies. The comments did not explain why the government would want to support this industry.

*“I guess big pharma felt left out during the recession heist…so now we need some way of handing over countless millions to them.”* [CBC August 6]

#### 2. Government protecting pharmaceutical industry

Commenters were suspicious about why the government was protecting pharmaceutical companies from legal recourse in the event of deleterious outcomes from the vaccine. The concern was that the companies knew or suspected that the vaccine was not safe and yet was releasing it for public use.

*“Hey Big Pharma, if this "novel split vaccine" is so wonderful and safe, why do you require such blanket protection from litigation?”* [GM October 23]

#### 3. Pharmaceutical companies benefit from the pandemic

Commenters seemed disturbed that pharmaceutical companies would be financially benefitting from a public health crisis. There appeared to be disdain for the windfall profits that commenters believed the companies would be earning as a result of selling millions of doses of vaccine to the government. Concern was also expressed that the companies were making decisions about the vaccine that were motivated by financial incentives rather than the public's best interest. For example, commenters wrote that the inclusion of the adjuvant would enable more doses to be sold and thus would lead to greater profits, and that the vaccine was being sold before adequate clinical trials were conducted in order to start selling their product.

*“Profit of course is the elephant in the room in this discussion. It is the real reason that Big Pharma and their Big Government friends are pushing the vaccine so vigorously and railing so angrily against the doubters.”* [GM October 23]

### Personal protective measures

Commenters proposed measures that people could take in order to protect themselves from H1N1 without getting vaccinated. These commenters believe that people have the ability to protect themselves from the virus. The alternatives to vaccination fell into two broad categories: basic prevention and diet/dietary supplements.

#### 1. Basic prevention

Commenters used their comments to remind or educate people about basic ways to prevent disease transmission and infection. Suggestions included calls for hand washing, coughing and sneezing into your sleeve, staying home when you're sick, staying several feet away from sick people, and drinking lots of fluids. Some comments shared educational information about how we become infected with viruses and how to prevent infection, such as not touching your eyes or nose.

*“God almighty - when did so many people become so incapable of dealing with anything? Wash your hands. Cough into your sleeve. Eat nutritious food. Drink plenty of water. Get plenty of rest. Stop shaking hands. Oh yah - homemade chicken broth is a good idea too. These things will save you. Not the crap that's been loaded into a syringe.”* [CBC August 6]

#### 2. Diet/dietary supplements

A proposed alternative to vaccination suggested by commenters was maintaining good health by eating a nutritious diet and/or using dietary supplements. Some commenters explicitly drew a causal connection between good nutrition and a boosted immune system that would enable individuals to ward off infections, including H1N1.

*“Eat healthy, take natural supplements and exercises regularly: I haven't caught a cold in years!”* [GM November 1]

### Frequency of themes over time

**Figure 1:**
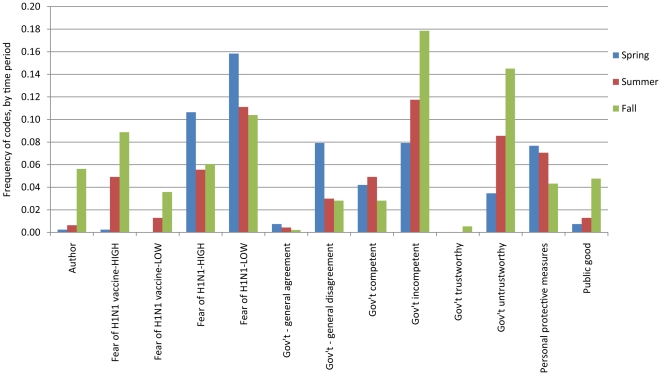
Coding by time period for themes with a consistent trend over time. Frequency of codes associated with the 8 themes that showed a consistent increase or decrease across time periods, by time period.

The frequency with which themes were mentioned in the comments fluctuated over time. Eight **themes** showed a consistent increase or decrease across time periods (spring, summer, fall). The frequency with which the **codes** associated with these 8 themes were applied in each period is presented in Figure 1. It is informative to see the change in codes, rather than just the change in the themes, because it gives a more complete picture about the changing relevance of topics to commenters. For example, with each subsequent time period, comments about fear of H1N1 decreased. Comments expressing high **and** low fear were mentioned less often over time, which suggests that commenters were less focused on the disease as the pandemic evolved. Conversely, commenters became more focused on the vaccine, with an increase in comments about high **and** low fear of the vaccine rising over time. The changes over time were not driven by a loss of interest in a topic by commenters with one perspective but rather the topic seems to have changed in relevance to commenters from both sides of the spectrum on an issue. The frequency of comments about government incompetency and untrustworthiness steadily increased over time, suggesting a growing focus on the government's role in the pandemic. Comments on government as trustworthy remained at or near zero throughout the pandemic, and comments on government competency fluctuated. Comments about vaccination as a public good and the authors of news stories increased over time, but remained relatively low in all time periods. There are confounding variables that may have impacted the trends over time, which are discussed in the study limitations.

### Frequency of themes by source

**Figure 2:**
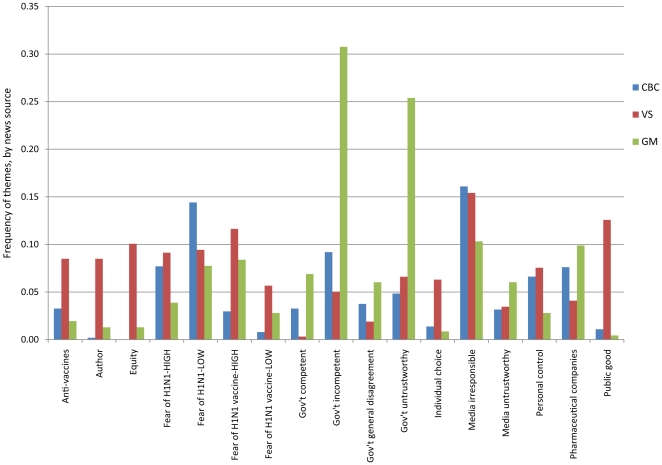
Themes by news source. Frequency of themes within each news source; includes themes associated with at least 5% of comments from at least one news source.

The frequency with which themes were mentioned in the comments varied across news sources. Figure 2 shows the frequency of themes by source for themes that constituted at least 5% of comments from at least one news source. Some of the greatest variation related to comments about the government. GM commenters were much more likely to comment on government incompetency and untrustworthiness than commenters from other sources. CBC commenters were more likely to mention a low fear of H1N1 and to comment less about fear (low or high) of the H1N1 vaccine compared to commenters from VS and GM. VS commenters were outliers for several themes; they were more likely to make comments related to equity, public good, individual choice, the article's author, and general opposition to vaccines as well as less likely to make comments about the government being competent. There are confounding variables that may have impacted the apparent variation by source, which are discussed in the study limitations.

### Validity

The comments posted in response to on-line news articles dealing with H1N1 and its vaccine revealed a high degree of consistency with findings from other studies, including pre-pandemic focus groups on attitudes about use of new vaccines in a pandemic and H1N1 surveys conducted during the 2009 pandemic. As in the comments, the focus groups found that people are concerned about compromised safety testing conducted during a pandemic and the relative risks of the disease versus the vaccine, emphasize personal protective measures that could be used in lieu of vaccination, and hold a mistrust of vaccines developed and tested by pharmaceutical companies [5]. Both the surveys and the comments showed that people were drawing comparisons between H1N1 and seasonal flu [9]–[11], [25] and that, in general, people had low fear of H1N1 but high fears about the safety of the H1N1 vaccine [6], [9]–[10], [26]–[27]. A survey of health care workers found that 48% of respondents who rejected the H1N1 vaccine said they preferred to use personal protective measures [28], which is consistent with the commenters who also planned to rely on such measures. Despite the similarities, we found discrepancies between the focus groups and the comments related to public good, individual choice and equity which received more attention in the focus groups than in the comments.

The similarities and differences across studies suggest that on-line comments can be an informative and important complementary source of information on the public's attitudes during health crises. It is valuable to have evidence supporting the importance of on-line comments as data because the comments are posted as events unfold (rather than respondents recollecting after the fact how they felt during an event) and the data are publicly and immediately available. The on-line comments can provide stand-alone data as well as provide data that complement surveys and other types of studies.

## Discussion

The format of on-line comments allows people to raise or *not* raise any topic they choose. This is a key element to understanding what impacts people's decision making on an issue as it allows us to identify not only what does matter to people but also what doesn't matter (or matters less) by the topics that are not commented upon. Themes mentioned infrequently can be assumed to be *less* relevant to people's decision making. This can have two implications. First, if health officials believe that these rarely mentioned themes are important then increased or improved communications may be necessary to bring them more fully into the public's consciousness. Second, if health communicators had believed that some of these themes mattered greatly to people (even though they were not considered critical from the health officials' perspective) and they had been addressing them in communications, then communicators can refocus away from these less salient and less important (from a public health perspective) topics and concentrate on the issues that matter most to the public and/or public health. For instance, based on the low frequency of comments about the public good of getting vaccinated, health communicators could increase messaging about the importance of getting vaccinated to help protect vulnerable people in the population and reduce rates of transmission and the spread of the disease.

The comments revealed a public that often views the government with mistrust about their motives in making health policies and recommendations and as incompetent to handle a health crisis (even if they have good intentions). This is problematic because the public will be less likely to accept government strategies, such as mass vaccinations, if their confidence in the government is low [29]–[30]. Similarly, media are viewed warily with coverage seen as lacking in depth and context that is necessary to empower the public to make informed decisions. There is also an element of mistrust, with some suspicions that the media hype stories in order to increase readership/viewership and consequently create a false sense of alarm. If the information conveyed by the media is deemed inadequate or mistrusted, then the public may be less likely to accept the messages.

The finding that H1N1 influenza was cognitively linked with seasonal flu indicates a potential problem for future health crises. It is likely that there will be other “flu” viruses that cause serious outbreaks or pandemics, and the public makes vaccination decisions largely based on weighing the perceived relative risks of the disease and the vaccine [11], [26], [31]–[33]. Given that large swaths of the public view seasonal flu as low risk and see a new vaccine as high risk, low vaccine uptake can be anticipated. This may be exacerbated by the relatively low number of deaths associated with H1N1, which may contribute to the belief that flus are not serious. On the other hand, in the event of an outbreak of a disease with high mortality rates, vaccines are likely to be more readily accepted because the risk of the disease will be perceived as greater than the risk of vaccination [34].

Despite the advantages that arise from using data that are completely driven by the respondent, using on-line comments is complicated by the role played by the news stories themselves. Although readers can comment on any aspect of an issue, it is reasonable to assume that the focus of comments and the ensuing conversation that develops among commenters will be influenced by the specific content of the news story to which the commenters are responding. A theme may be frequently mentioned in a series of comments *because* that theme relates to the topic of the story. This raises a question of whether a frequently mentioned theme is mentioned because it is important to the commenter or because it corresponds to the article's topic. In fact, we believe it is the former. We believe it can be assumed that readers choose to post a comment because they feel that an issue is important and they have a strong reaction or opinion related to the issue. A particular news story may create an outlet for the reader to express his opinion but the story is less likely to cause a reader to develop passion for an issue for which he previously lacked passion. Hence, when readers post comments on a theme that is also the theme of an article, the article is providing a forum for expressing opinions on a topic that resonated with the reader. In our study, we included articles that received the most comments. This screening strategy allowed us to select stories that resonated most strongly with readers (assuming that readers post comments preferentially in response to those stories for which they have the strongest feelings). Consequently, even though a theme may get mentioned often because it relates to the article's theme, we are still identifying the themes that matter most to the commenters because they would not be posting comments (or far fewer comments) if that theme was of low importance.

One could also argue that readers are more likely to post comments if they disagree with a news story or feel discontent about an issue, thus biasing our understanding of public opinions. It is the case that for all of our themes that could have a positive or negative response, negative comments were more common than positive comments. However, it is not clear whether this is because people who felt positively about an issue were less likely to comment or because there are fewer people who feel positive. For example, did few people express a belief that the government was competent to handle the pandemic because few people believe this or because those who think the situation is being well handled did not feel motivated to comment? There is reason to believe that people with opinions on both ends of a topic's spectrum do post comments. For example, on the issues of the severity of H1N1 and concerns about the H1N1 vaccine, significant numbers of comments expressing both low and high fear of the disease and the vaccine were expressed. Determining the extent to which comments may be biased by negative comments requires further study.

The comments offer some insight into changes in the topics that people were commenting on over the course of the pandemic and variability in perspectives by commenters of different news sources. These trends should be interpreted cautiously because the theme frequencies may be biased by unequal representation from each news source across time periods and from articles addressing topics related to a particular theme.

Our study has two key limitations. First, no demographic information is available with the comments so we are unable to assess the representativeness of the comments. Second, the frequency with which each theme was mentioned could be biased in two ways: 1) there was not an equal number of comments from each news source which could create a bias if there are differences among commenters related to source; and 2) the themes mentioned in the comments can be related to the content of the article to which the commenters are responding. We believe this limitation is mitigated by including comments from 12 articles that cover many topics related to H1N1 vaccine and different time periods during the pandemic.

The strengths of our study include the large sample size (1,796 comments), that comments were posted in real-time so data reflect how people felt at the time that they read about a news event rather than recollecting their perceptions and emotions, and that comments capture whatever the commenters felt was important to mention rather than being constrained by questions posed by researchers.

The insights derived from the comments about the public's perspectives and attitudes can contribute to improved communication about health crises in general and new vaccines in particular, allowing for more effective communication about established health recommendations as well as informing dialogue between health officials and the public and enabling policy and practice decision making that incorporates a broader range of views.

Some key issues that appear important to address in communications related to new vaccines before and during a health crisis include:

- Providing information about the pharmaceutical industry: how it is regulated and the nature of the relationship between pharmaceutical companies and the government (or clarification about an absence of a relationship);
- Providing balance in messages about the value of personal protective measures as well as the limited effectiveness of these steps and, consequently, the need for vaccination;
- Correcting misperceptions about how vaccine safety testing is conducted during a pandemic and addressing concerns about inadequate sample size and time to detect long term effects;
- Increasing messaging about the risks and seriousness of the illness so that sufficient risk is attributed to it (this is especially true for flus that are classified cognitively with seasonal flu – an illness that is generally perceived as low risk).

Some key issues that appear important to address in policy and practice decision making for dealing with health crises include:

- Forming partnerships for vaccine development between pharmaceutical companies and more trusted institutions, such as universities;
- Improving the public's perceptions of government trustworthiness and competence;
- Establishing guidelines or strategies within media sources such that the public perceives news coverage as trustworthy, thorough and accurate;
- Creating plans for communication using non-traditional channels of dissemination (i.e., other than mainstream media).
- Recognizing that the public are not passive recipients of messages from public health officials, but have strong and valid opinions about health care and health issues. There is an opportunity for engaging in dialogue with the general public that can inform all parties and contribute towards improving health communications [17].

### Conclusion

Comments posted by readers of articles related to the H1N1 vaccine on three Canadian news websites revealed seven themes that appear to be most important to the commenters and which, consequently, may have played a significant role in their decision making about whether or not to receive the H1N1 vaccine. Using on-line comments may be a valuable new source of insights into public opinions and can be used to assess public perspectives in real time.

## References

[1] Chan M (2009). World now at the start of 2009 influenza pandemic.. http://www.who.int/mediacentre/news/statements/2009/h1n1_pandemic_phase6_20090611/en/index.html.

[2] TheCanadianPress (2009). Canadians grow more concerned about H1N1, more plan to get shot: poll.. http://ca.entertainment.yahoo.com/s/capress/091103/national/flu_poll_1.

[3] StatisticsCanada (2010). Canadian Community Health Survey: H1N1 vaccinations.. http://www.statcan.gc.ca/daily-quotidien/100719/dq100719b-eng.htm.

[4] Ritvo P, Irvine J, Klar N, Wilson K, Brown L (2003). A Canadian national survey of attitudes and knowledge regarding preventive vaccines.. J Immune Based Ther Vaccines.

[5] Henrich N, Holmes B (2009). The public's acceptance of novel vaccines during a pandemic: a focus group study and its application to influenza H1N1 Emerging Health Threats Journal.

[6] Sypsa V, Livanios T, Psichogiou M, Malliori M, Tsiodras S (2009). Public perceptions in relation to intention to receive pandemic influenza vaccination in a random population sample: evidence from a cross-sectional telephone survey.. Euro Surveill.

[7] Eastwood K, Durrheim D, Jones A, Butler M (2010). Acceptance of pandemic (H1N1) 2009 influenza vaccination by the Australian public.. Med J Aust.

[8] Maurer J, Uscher-Pines L, Harris KM (2010). Perceived seriousness of seasonal and A(H1N1) influenzas, attitudes toward vaccination, and vaccine uptake among U.S. adults: Does the source of information matter?. Preventive Medicine.

[9] Schwarzinger M, Flicoteaux R, Cortarenoda S, Obadia Y, Moatti J-P (2010). Low Acceptability of A/H1N1 Pandemic Vaccination in French Adult Population: Did Public Health Policy Fuel Public Dissonance?. PLoS ONE.

[10] Seale H, Heywood A, McLaws M, Ward K, Lowbridge C (2010). Why do I need it? I am not at risk! Public perceptions towards the pandemic (H1N1) 2009 vaccine.. BMC Infect Dis.

[11] Setbon M, Raude J (2010). Factors in vaccination intention against the pandemic influenza A/H1N1.. Eur J Public Health.

[12] Van D, McLaws M, Crimmins J, MacIntyre C, Seale H (2010). University life and pandemic influenza: Attitudes and intended behaviour of staff and students towards pandemic (H1N1) 2009.. BMC Public Health.

[13] Wong LP, Sam IC (2010). Factors influencing the uptake of 2009 H1N1 influenza vaccine in a multiethnic Asian population.. Vaccine.

[14] Sinuff T, Cook D, Giacomini M (2007). How qualitative research can contribute to research in the intensive care unit.. J Crit Care.

[15] Sandelowski M (2004). Using qualitative research.. Qual Health Res.

[16] Internet World Stats (2010). Internet usage statistics.. http://www.internetworldstats.com/stats.htm.

[17] Green L, Ottoson J, Garcia C, Hiatt R (2009). Diffusion theory and knowledge dissemination, utilization, and integration in public health.. Annu Rev Public Health.

[18] Manosevitch E, Walker D (2009). Readers comments to online opinion journalism: a space of public deliberation..

[19] Rowe G, Hawkes G, Houghton J (2008). Initial UK public reaction to avian influenza: Analysis of opinions posted on the BBC website.. Health, Risk & Society.

[20] Poria Y, Oppewal H (2003). A new medium for data collection: online news discussions.. International Journal of Contemporary Hospitality Management.

[21] Shanahan M-C (2010). Changing the meaning of peer-to-peer? Exploring online comment spaces as sites of negotiated expertise.. JCOM: Journal of Science Communication.

[22] Sooyoung C, Youngshin H (2007). Netizens' Evaluations of Corporate Social Responsibility: Content Analysis of CSR News Stories and Online Readers' Comments..

[23] Wright N, Nerlich B (2006). Use of the deficit model in a shared culture of argumentation: The case of foot and mouth science.. Public Understanding of Science.

[24] Lupton D (1992). Discourse analysis: A new methodology for understanding the ideologies of health and illness.. Australian Journal of Public Health.

[25] Poland GA (2010). The 2009-2010 influenza pandemic: effects on pandemic and seasonal vaccine uptake and lessons learned for seasonal vaccination campaigns.. Vaccine.

[26] SteelFisher GK, Blendon RJ, Bekheit MM, Lubell K (2010). The Public's Response to the 2009 H1N1 Influenza Pandemic.. New England Journal of Medicine.

[27] Nougairède A, Lagier J-C, Ninove L, Sartor C, Badiaga S (2010). Likely Correlation between Sources of Information and Acceptability of A/H1N1 Swine-Origin Influenza Virus Vaccine in Marseille, France.. PLoS ONE.

[28] Kaboli F, Astrakianakis G, Li G, Guzman J, Naus M (2010). Influenza Vaccination and Intention to Receive the Pandemic H1N1 Influenza Vaccine among Healthcare Workers of British Columbia, Canada: A Cross-Sectional Study.. Infection Control and Hospital Epidemiology.

[29] Chanley V, Rudolph T, Rahn W (2000). The Origins and Consequences of Public Trust in Government: A Time Series Analysis Public Opinion Quarterly.

[30] Gutteling J, Hanssen L, van der Veer N, Seydel E (2006). Trust in governance and the acceptance of genetically modified food in the Netherlands Public Understanding of Science.

[31] Rhodes S, Hergenrather K (2003). Using an integrated approach to understand vaccination behavior among young men who have sex with men: Stages of change, the health belief model and self efficacy.. Journal of Community Health.

[32] Editorial (2009). How to win trust over flu.. Nature.

[33] Fu F, Rosenbloom DI, Wang L, Nowak MA (2010). Imitation dynamics of vaccination behaviour on social networks..

[34] Brewer N, Chapman G, Gibbons F, Gerrard M, McCaul K (2007). Meta-analysis of the relationship between risk perception and health behavior: the example of vaccination.. Health Psychology.

